# Supplementary material for: Information access and COVID-19 vaccination hesitancy among foreign-born persons in Sweden – a focus group interview-study
Source: BMC Public Health. 2024 Dec 5;24:3389. doi: 10.1186/s12889-024-20959-y (PMC11622565; doi:10.1186/s12889-024-20959-y)
Supplement: Supplementary file 1 — Supplementary Material 1 [file 12889_2024_20959_MOESM1_ESM.pdf]

Appendix I

Examples of open-ended questions, used as prompts in the focus group interviews.

What sources have you used to obtain information about COVID-19?
Which information sources felt most / least reliable?
How did you find out that a COVID-19 vaccine was available in Sweden?
How did you feel about taking the vaccine?
What affected your decision to take the vaccine?
How can information be improved to make people more interested in taking the vaccine?
